# Supplementary material for: Identification of Novel Genomic Associations and Gene Candidates for Grain Starch Content in Sorghum
Source: Genes (Basel). 2020 Dec 2;11(12):1448. doi: 10.3390/genes11121448 (PMC7760202; doi:10.3390/genes11121448)

Identification of novel genomic associations and gene candidates for grain starch content in sorghum

Sirjan Sapkota[1,2,*] , J. Lucas Boatwright[2], Kathleen Jordan[2], Richard Boyles[1,3], and Stephen Kresovich[1,2],

1 Department of Plant and Environmental Sciences, Clemson University, Clemson, SC, USA
2 Advanced Plant Technology Program, Clemson University, Clemson, SC, USA
3 Pee Dee Research and Education Center, Clemson University, Florence, SC, USA

* correspondence: ssapkot@g.clemson.edu

Supplementary File

Table S1. Variance components for the linear mixed models fit using genetic and environmental variables.

	Geno	Year	Geno × Year	Year × Rep	Residual	Total	H^2
Starch	3.364	0.000	1.070	0.141	2.922	7.497	0.799

Table S2. Pairwise linkage disequilibrium between associated SNPs and their neighboring SNPs. R^2: correlation coefficient, Chr: Chromosome. SNP: single nucleotide polymorphism.

Chr SNP1	Position SNP1	Chr SNP2	Position SNP2	R^2
1	4067535	1	4063988	0.003
1	4067535	1	4067364	0.873
1	4067535	1	4067377	0.873
1	4067535	1	4067389	0.346
1	4067535	1	4067441	0.067
1	4067535	1	4067503	0.068
1	4067535	1	4067535	1.000
1	4067535	1	4067555	1.000
1	4067535	1	4067562	0.078
1	4067535	1	4067566	0.674
1	4067535	1	4068079	0.184
1	4067535	1	4070689	0.022
8	51715166	8	51695627	0.057
8	51715166	8	51715166	1.000
8	51715166	8	51719632	0.219
8	51715166	8	51719659	0.225
8	51715166	8	51719688	0.216
8	51715166	8	51719704	0.993
8	51715166	8	51720767	0.938
8	51715166	8	51721062	0.816
8	51715166	8	51721065	0.794
8	51715166	8	51726098	0.812
8	51715166	8	51727032	0.061
8	59121722	8	59121680	0.146
8	59121722	8	59121722	1.000
8	59121722	8	59121748	0.116
8	59121722	8	59121837	0.119
8	59121722	8	59121843	0.989
8	59121722	8	59136112	0.973
8	59121722	8	59136162	0.969
8	59121722	8	59136258	0.828
8	59121722	8	59136260	0.988
8	59121722	8	59136281	0.810
8	59121722	8	59136549	0.957
8	59121722	8	59136555	0.957
8	59121722	8	59141038	0.216
8	59121722	8	59141815	0.093

Table S3. KEGG Pathway and UniProt keywords enrichment results for gene network of HSP 90-6 gene, Sobic.008G111600. FDR: false discovery rate, Observed and Expected refer to the gene count for the network. FDR Threshold: -log10(0.01) = 2.

	**Term ID**	**Term description**	**Observed gene count**	**Background gene count**	**FDR** [-log10(p)]
KEGG Pathway	sbi04141	Protein processing in endoplasmic reticulum	16	200	18.680
	sbi03018	RNA degradation	7	109	7.444
	sbi04144	Endocytosis	6	150	5.359
	sbi04626	Plant-pathogen interaction	6	162	5.297
	sbi03040	Spliceosome	6	189	5.020
UniPort keywords	KW-0802	TPR repeat	22	91	35.614
	KW-0040	ANK repeat	14	115	18.583
	KW-0067	ATP-binding	14	1053	6.312
	KW-0175	Coiled coil	25	3633	6.243

Table S4. Summary of significant epistatic interaction between associated variants and variants within genes that show high protein-protein interaction with heat shock protein 90-6 (*Sobic.008G111600*). Chr: chromosome, β_{INT}: regression coefficient of interaction.

Chr	Position	Chr	Position	β_{INT}	Stat	-log10(p)	Gene
8	51715166	3	6644300	-0.71	20.25	5.167	Sobic.003G077800
8	51715166	4	172893	-0.77	18.61	4.795	Sobic.004G002200
8	51715166	4	173153	-0.74	17.54	4.550	Sobic.004G002200
8	51715166	4	64477961	0.43	19.57	5.013	Sobic.004G306700
8	51715166	4	64478176	0.38	15.41	4.063	Sobic.004G306700
8	51719704	3	6644300	-0.70	19.28	4.946	Sobic.003G077800
8	51719704	4	172893	-0.77	18.28	4.719	Sobic.004G002200
8	51719704	4	173153	-0.75	17.23	4.479	Sobic.004G002200
8	51719704	4	64477961	0.44	20.63	5.253	Sobic.004G306700
8	51719704	4	64478176	0.38	15.44	4.068	Sobic.004G306700
8	51720767	1	69932728	0.79	22.43	5.661	Sobic.001G418600
8	51720767	3	6644300	-0.65	17.69	4.584	Sobic.003G077800
8	51720767	4	172893	-0.72	17.09	4.447	Sobic.004G002200
8	51720767	4	173153	-0.69	15.71	4.130	Sobic.004G002200
8	51720767	4	64477961	0.45	19.30	4.951	Sobic.004G306700
8	51720767	4	64478169	0.39	15.73	4.136	Sobic.004G306700
8	51720767	4	64478176	0.40	16.59	4.333	Sobic.004G306700
8	51720767	4	64478528	-0.37	15.62	4.111	Sobic.004G306700
8	51721065	1	69932728	0.89	26.41	6.557	Sobic.001G418600
8	51721065	3	6644300	-0.66	16.81	4.383	Sobic.003G077800
8	51721065	4	172893	-0.72	16.03	4.206	Sobic.004G002200
8	51721065	4	173153	-0.70	15.31	4.039	Sobic.004G002200
8	51721065	4	64477961	0.43	16.50	4.313	Sobic.004G306700
8	51721065	10	7353746	-1.00	21.21	5.385	Sobic.010G085600
8	51726098	1	69932728	0.85	25.29	6.307	Sobic.001G418600
8	51726098	3	6644300	-0.67	17.59	4.561	Sobic.003G077800
8	51726098	4	172893	-0.73	16.67	4.351	Sobic.004G002200
8	51726098	4	173153	-0.70	15.29	4.035	Sobic.004G002200
8	51726098	4	64477961	0.43	16.81	4.384	Sobic.004G306700
8	51726098	4	64478169	0.40	16.01	4.199	Sobic.004G306700
8	51726098	4	64478176	0.42	17.21	4.475	Sobic.004G306700
8	51726098	4	64478528	-0.39	16.52	4.317	Sobic.004G306700
8	51726098	10	7353746	-1.00	21.62	5.477	Sobic.010G085600

Table S5. Null distribution of mean posterior inclusion probability (PIP) values from ten simulated data sets across quantiles to determine the empirical significance cutoff for associations from the Bayesian sparse linear mixed model (BSLMM).

Quantile	0%	25%	50%	75%	95%	99%	99.9%	99.99%	100%
PIP	0.0001	0.0004	0.0006	0.0008	0.0012	0.0021	0.0061	0.0300	0.0658

Figure S1. Genes within the neighborhood of heat shock protein 90-6, *Sobic.008G111600*. Highlighted in blue are genes: *Sobic.008G111300* and *Sobic.008G111100*. Figure obtained from Gene Tree Viewer in Gramene (www.gramene.org).

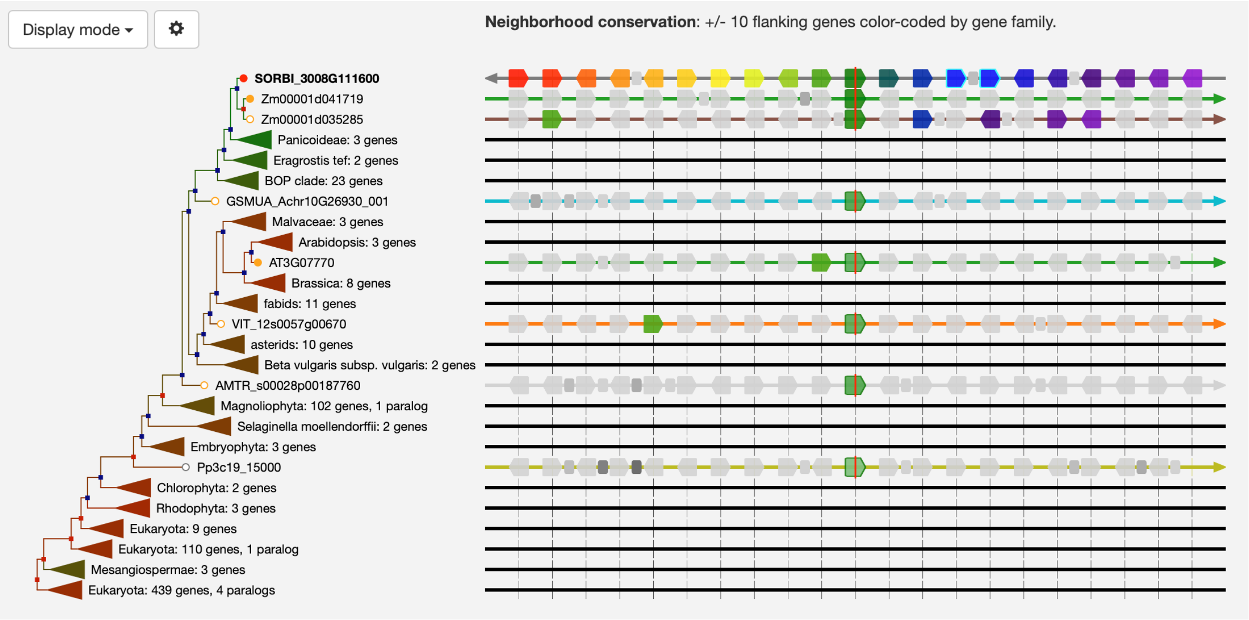

Figure S2. Gene expression profile of the seven paralogs of heat shock protein 90 in sorghum. Y-axes represent transcript abundance in transcript per million (TPM) and x-axes corresponds to tissue type. infl: infloresence, emer: post-emergence, DAP: days after pollination.

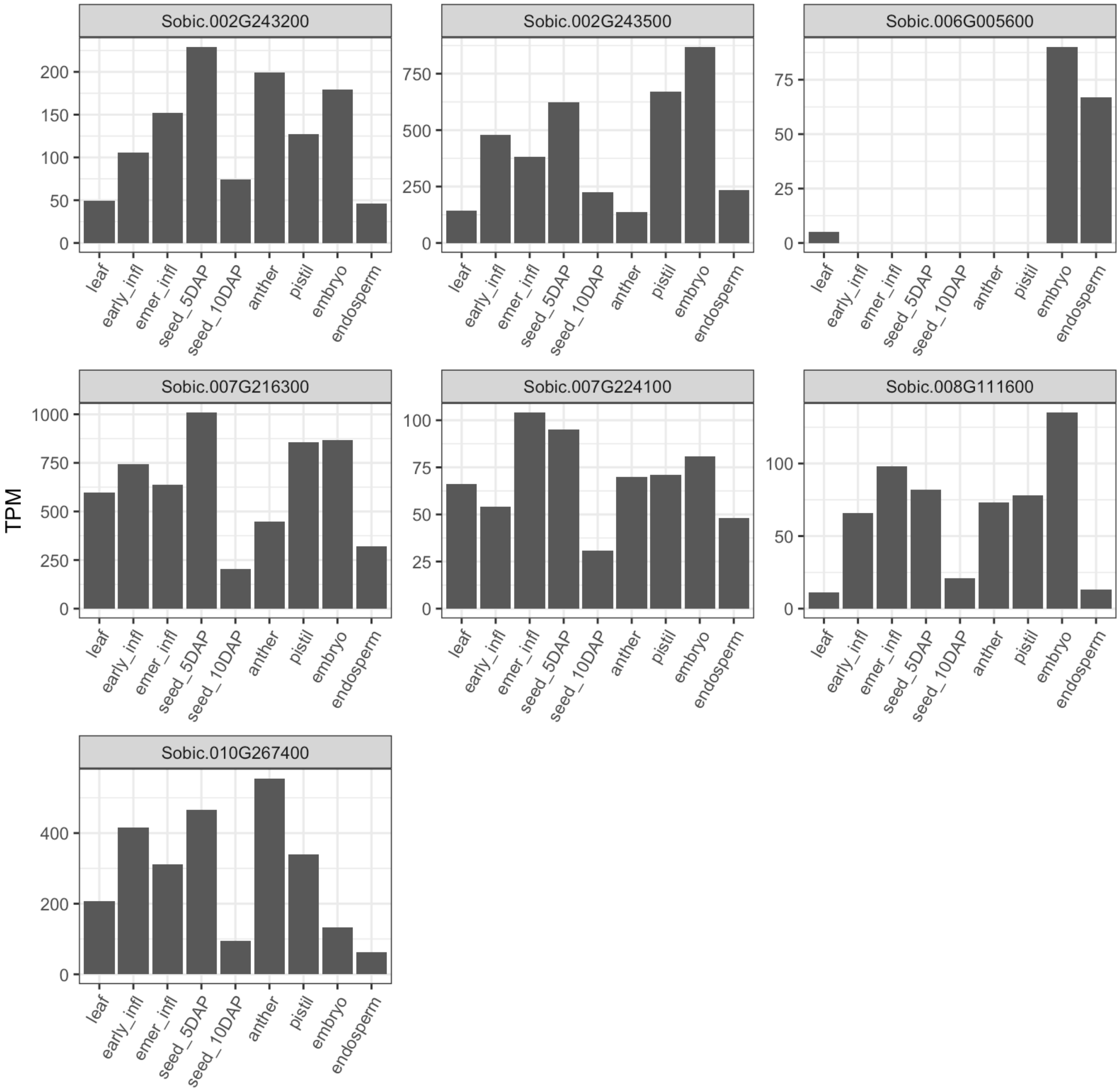

Supplement: Supplementary file 1 [file genes-11-01448-s001.pdf]
